# Revisiting Host-Binding Properties of LigA and LigB Recombinant Domains

**DOI:** 10.3390/microorganisms13061293

**Published:** 2025-05-31

**Authors:** Henrique M. Pires, Igor R. M. Silva, Aline F. Teixeira, Ana L. T. O. Nascimento

**Affiliations:** 1 Laboratório de Desenvolvimento de Vacinas, Instituto Butantan, Avenida Vital Brazil, São Paulo 05503-900, SP, Brazil; h.pires.proppg@proppg.butantan.gov.br (H.M.P.); i.rsilva.proppg@proppg.butantan.gov.br (I.R.M.S.); aline.rteixeira@fundacaobutantan.org.br (A.F.T.); 2 Programa de Pós-Graduação Interunidades em Biotecnologia, Instituto de Ciências Biomédicas, Universidade de São Paulo, São Paulo 05508-900, SP, Brazil

**Keywords:** *Leptospira*, leptospirosis, LigA, LigB, host interactions

## Abstract

Pathogenic bacteria of the genus *Leptospira* are the etiological agents of leptospirosis, a disease that affects humans and animals worldwide. Despite the increasing number of studies, the mechanisms of leptospiral pathogenesis remain poorly comprehended. In this study, we report various interactions of the LigA7’-13’ and LigB1’-7’ domains with host components. The LigA7’-13’ and LigB1’-7’ were cloned into the pET28a vector, and the recombinant proteins were expressed in *E. coli* C43 (DE3) and *E. coli* BL21 (DE3), respectively. Both recombinant protein domains were expressed in soluble form and purified using nickel-chelating chromatography. The rLigA7’-13’ and rLigB1’-7’ domains exhibited binding to several types of integrins, with most interactions occurring in a dose-dependent and saturable manner, consistent with the characteristics of typical receptor-ligand interactions. The recombinant domain LigA7’-13’ demonstrated affinity for the glycosaminoglycans (GAGs) chondroitin-4-sulfate, chondroitin sulfate, heparin, chondroitin sulfate B, and heparan sulfate, while no binding was detected for LigB1’-7’ with these molecules. Both rLigA7’-13’ and rLigB1’-7’ interacted with components of the terminal complement pathway and were capable of recruiting C9 from normal human serum (NHS). These interactions may inhibit the formation of polyC9, ultimately preventing the assembly of the membrane attack complex (MAC). Collectively, our data expand the repertoire of host components that interact with rLigA7’-13’ and rLigB1’-7’, opening new avenues for understanding leptospiral immune evasion and broadening the roles of these domains in bacterial virulence.

## 1. Introduction

Leptospirosis is a widespread zoonosis, predominantly found in subtropical and tropical regions, caused by pathogenic bacteria of the genus *Leptospira*. The widespread distribution of leptospires can be attributed to their capacity to colonize the renal tubules of a variety of mammalian hosts. Some of these hosts serve as reservoir species that exhibit no clinical signs of disease, yet the bacteria persist in their kidneys, shedding viable leptospires in their urine and contaminating the environment [1,2]. The bacteria can penetrate the host through intact, damaged, or wet skin and/or mucous membranes [3]. The symptoms of leptospirosis are nonspecific and flu-like, including fever and headache, often leading to misdiagnosis as other febrile illnesses. In some instances, human leptospirosis can progress to more severe manifestations, such as hemorrhagic syndromes (pulmonary hemorrhage syndrome), hypotension, and multiple organ failure, collectively referred to as Weil’s disease [1,4,5]. In developing countries, the incidence of human leptospirosis is linked to poor sanitary conditions, while in developed nations, it is related to recreational events such as canoeing or occupational exposures [6,7]. Additionally, leptospirosis has significant economic implications as it affects livestock, leading to reduced growth rates, abortion, decreased milk production, and mortality [1,2].

The contact of pathogens with the extracellular matrix (ECM) plays a critical role in the colonization of host tissues [8]. Pathogens, including spirochetes, have been shown to subvert host proteases, such as plasmin, to achieve proteolytic activity during infection [9]. The fibrinolytic system is capable of degrading the ECM, thereby facilitating bacterial invasion [9]. Once in the bloodstream, bacteria must evade the host’s immune response to reach target organs. Several leptospiral proteins, expressed as recombinant proteins, have demonstrated the ability to bind ECM molecules and plasminogen, including the LigA and LigB domains [10,11].

Immunoglobulin-like domain proteins, specifically LigA and LigB, were first described in 1973 as Fab fragments of human immunoglobulin [12] and are exclusively present in pathogenic *Leptospira*. Evidence suggests that both LigA and LigB are associated with the pathogenicity of *L. interrogans* [13]. For this reason, the LigA and LigB proteins are considered promising candidates for the development of subunit vaccines against leptospirosis. Numerous studies have investigated immunoprotection using either recombinant Lig proteins or *lig* DNA, and the collected data have been recently reviewed [14]. The LigA7’-13’ domain, alone or in association with LigB1’-7’, demonstrated immunoprotection in a hamster model challenged with virulent *L. interrogans*. However, it is noteworthy that in this study, no bacteria were recovered from the kidneys, while residual leptospires were observed in other studies [15,16,17]. Furthermore, these proteins also serve as potential candidates for the serodiagnosis of leptospirosis [18]. The involvement of LigA and LigB proteins in the adhesion and invasion processes of pathogenic *Leptospira* has been explored by their interactions with laminin, collagen, elastin, tropoelastin, plasma and cellular fibronectin, fibrinogen, and plasminogen [19,20,21]. In addition, it has been shown that these proteins also interact with the complement system regulators Factor H (FH) and C4b binding protein (C4BP), which can block activation of alternative and classical pathways and contribute to leptospiral immune evasion [22].

Integrins and GAGs serve as cell adhesion receptors and components of the ECM, respectively, playing crucial roles in pathogenesis [23,24,25,26]. The capability of pathogenic leptospires to bind to cadherins, integrins, and GAGs has been previously documented [27,28,29]. To date, only a limited number of proteins have been identified as integrin- and GAG-binding [29,30,31,32].

In this study, we investigated various aspects of the host interactions of recombinant LigA7’-13’ and LigB1’-7’ domains by assessing their binding capabilities with integrins, GAGs, and components of the terminal steps of the complement system. The results indicate that these domains are capable of interacting with these host components, thereby revealing novel insights into the potential pathological mechanisms during the infection process.

## 2. Materials and Methods

### 2.1. Biological Components

The macromolecules chondroitin sulfate (shark cartilage, C4384), chondroitin sulfate B (porcine intestinal mucosa, C3788), chondroitin-4-sulfate (bovine trachea, 27042), heparin (porcine intestinal mucosa, H4784), heparan sulfate (bovine kidney, H7640), fetuin (fetal serum bovine, F3385), and BSA (bovine serum albumin, A7906) were purchased from Sigma-Aldrich, St. Louis, MO, USA. C5b,6 (human, A122), C6 (human, A123), C7 (human, A124), C8 (human, A125), and C9 (human, A126) were purchased from Complement Technology (USA). Integrins α8, αVβ3, αVβ5, α5β1, αVβ6, αVβ8, αLβ2, αIIbβ3, αMβ2, αVβ1 were purchased from R&D Systems (MN, USA). The commercial antibodies used were peroxidase (HRP)-conjugated anti-mouse IgG (A9044) and mouse monoclonal anti-polyhistidine-peroxidase antibody (A7058) acquired from Sigma-Aldrich, St. Louis, MO, USA.

### 2.2. Cloning, Expression, and Purification of rLigA7’-13’ (625-1224) and rLigB1’-7’ (131-645) Recombinant Proteins

The sequences were amplified by PCR, without signal peptide, and subsequently cloned into the pET-28a vector at the restriction sites NdeI-XhoI and SacI-XhoI for rLigA7’-13’ and LigB1’-7’, respectively. The cloned sequences were verified by automated sequencing (3500 Genetic Analyzer, Applied Biosystems—Auburn, AL, USA). *E. coli* C43 (DE3) was transformed with the pET-28a-rLigA7’-13’ plasmid, while *E. coli* BL21 (DE3) was transformed with the pET-28a-rLigB1’-7’. These cells were grown at 37 °C in Luria-Bertani (LB) liquid medium supplemented with kanamycin, and the recombinant proteins were induced by the addition of 1 mM IPTG (isopropyl β-D-1-thiogalactopyranoside). Bacterial cultures were centrifuged at 6000× *g* for 10 min at 4 °C, and the pellets were resuspended in lysis buffer (20 mM Tris-HCl pH 8; 150 mM NaCl; 200 µg/mL lysozyme (Sigma-Aldrich); 2 mM PMSF (phenylmethanesulfonyl fluoride, Sigma-Aldrich); 1% Triton X-100). Cells were disrupted using a high-pressure homogenizer (Panda Plus 2000, GEA Group—Parma, Italy). The samples were centrifuged again at 10,000× *g* for 10 min at 4 °C, and the supernatants containing the soluble protein fractions were subjected to purification by nickel-affinity chromatography (AKTA-prime plus, Cytiva—Marlborough, MA, USA). The rLigA7’-13’ and rLigB1’-7’ proteins were eluted with ~20–40 mM imidazole and dialyzed against PBS at 4 °C for 24 h in three cycles, then stored at −20 °C. The purified fragments were evaluated by SDS-PAGE (12%).

### 2.3. Antiserum Production

BALB/c mice (18–22 g), with five animals per cage, were inoculated subcutaneously with 10 μg of each recombinant protein adsorbed in 12.5% Al(OH)_3_, followed by two booster doses approximately every 15 days. The animals were bled via the retro-orbital plexus prior to inoculation as a non-immune control and after each immunization. The collected blood was kept at 37 °C for 30 min and at 4 °C for 30 min. The clot was separated by centrifugation at 2500× *g* for 15 min at 4 °C, with the serum stored at −20 °C. The sera were analyzed using ELISA, where 250 ng of each recombinant protein was immobilized in wells for 16 h at RT. The wells were then blocked with a PBS-0.05% Tween 20 (PBS-T) solution containing 10% skimmed milk. Animal sera at varying serial dilutions were added to the wells and incubated for 1 h. After washing, an anti-mouse IgG secondary antibody conjugated to peroxidase was added. Reactivity was assessed by adding 1 mg/mL OPD (o-phenylenediamine) along with 1 μL/mL H_2_O_2_. After 10 min, the reaction was halted by adding 50 μL of 2 M H_2_SO_4_, and readings were taken at 492 nm using a Multiskan-FC microplate reader (Thermo Fisher Scientific, Helsinki, Finland). The antibody titer was determined as the inverse of the dilution that yielded an absorbance value of 0.1 at 492 nm. Blank controls without serum were included in all experiments, and three independent experiments were conducted.

### 2.4. Binding of Recombinant Fragments to Human Integrins and to GAGs

Each integrin was immobilized onto ELISA plates (100 nanograms per well) at 4 °C for 16 h, according to a previously described protocol with modifications [30]. After incubation with 1 µg of recombinant fragments, the plates were incubated at 37 °C for 1 h with polyclonal antibodies specific to each fragment (1:10,000), followed by incubation for 1 h with anti-mouse IgG (1:10,000). The reaction was revealed as described above. Gelatin was included as a negative control. Each GAG component was immobilized onto ELISA plates (100 µg per well for chondroitin-4-sulfate, chondroitin sulfate, and chondroitin sulfate B; 1 µg per well for heparin) at 4 °C for 16 h, following the protocol described above with some modifications. BSA was included as a negative control. After incubation with 1 µg of recombinant fragments, the plates were fixed with 2% paraformaldehyde at pH 7.4 for 30 min at RT [33]. The plates were then washed and incubated with 2% glycine for 30 min at RT. The reaction was revealed as previously described.

### 2.5. The Interaction Between rLigA7’-13’ and rLigB1’-7’ and Human Complement Components

Each component of the terminal complement system (C6, C7, C8, C9, C5b,6) was immobilized onto ELISA plates (1 µg per well) for 16 h at 4 °C. Fetuin (1 µg per well) was included as a negative control. Plates were washed with PBS-0.05% Tween 20 (PBS-T) to remove unbound components and blocked with PBS-T containing 10% nonfat dry milk (*w*/*v*) (PBS-T-Milk) for 1 h at 37 °C. Recombinant fragments (1 µg per well) were added, followed by incubation for 2 h at 37 °C. After three washes, the reaction of recombinant proteins with the components was detected by the addition of HRP-conjugated anti-His tag mAbs (1:10,000 *v*/*v*), followed by incubation for 1 h at 37 °C. Plates were washed six times with PBS-T, and binding was revealed using 1 mg/mL OPD (o-Phenylenediamine) in citrate-phosphate buffer at pH 5 along with 1 µL/mL H_2_O_2_. The plates were kept in the dark for 10 min, and the reaction was halted by the addition of 4 N H_2_SO_4_. Readings were taken at 492 nm using a Multiskan-FC microplate reader. The mean absorbance of each component with the recombinant protein was compared with the negative control using Student’s *t*-test, and a *p*-value below 0.05 was considered statistically significant (GraphPad Prism v8.07, Boston, MA, USA).

### 2.6. Dose Response Analysis of Recombinant Protein Fragments and the Host Components

The components that showed substantial interaction with the recombinant protein domains were immobilized on 96-well ELISA plates, as described above. Following blocking, increasing concentrations of recombinant proteins were added, and the reaction mixture was incubated for 2 h at 37 °C. For the GAGs dose-response assay, two additional steps were performed: fixation with paraformaldehyde and incubation with glycine. Subsequent incubation with a mouse antibody and detection of bound proteins were carried out as previously reported. The KD values and dose-response curves were determined using the “Non-linear Regression” tool in GraphPad Prism (version 8), applying a saturation binding model with “one site-specific binding.” Statistical differences were assessed using the Student’s *t*-test, comparing protein binding to the control.

### 2.7. Characterization of rLigA7’-13’ and rLigB1’-7’ Binding to C9

The interaction of rLigA7’-13’ and rLigB1’-7’ with C9 from NHS was assessed by immobilizing the recombinant proteins in well plates and incubating them with increasing dilutions of NHS. The binding was verified by the addition of goat anti-human C9 (1:10,000), followed by HRP-conjugated anti-goat IgG (1:50,000). As a negative control, BSA was used. The effect of rLigA7’-13’ and rLigB1’-7’ on C9 polymerization was evaluated according to a previously published protocol [31]. Briefly, in 20 mM Tris pH 7.4 with 0.15 M NaCl, 3 µg of C9 (Complement Technology, Tyler, TX, USA) was incubated with rLIC13259 (5 µg, positive inhibition control) [30], fetuin (5 µg, negative inhibition control), or rLigA7’-13’/rLigB1’-7’ (1.25, 2.5, and 5 µg) at 37 °C for 40 min. Then, 50 µM of ZnCl2 was added to mixture and an incubation of 2 h at 37 °C was performed to allow C9 polymerization. For reaction control, free C9 was incubated with or without the addition of ZnCl2. Samples were then analyzed by 4–20% gradient SDS-PAGE (Bio-Rad, Hercules, CA, USA), and detection of poly-C9 was performed using Coomassie blue staining.

### 2.8. Ethics Statement

This study was performed according to the guidelines outlined by the Brazilian National Council for Control of Animal Experimentation (CONCEA). It follows international guidelines for animal welfare and the principles of the 3Rs. Experimental protocols comply with the ARRIVE guidelines and were approved by the Ethics Committee on Animal Use of the Butantan Institute, São Paulo, Brazil (protocol no. 6068070224). Mice were housed in a BSL1 animal facility, in micro isolators with individual ventilation and temperature, and light cycle control. Animals received food and water ad libitum, and manipulation was performed by trained personnel.

## 3. Results

### 3.1. Cloning, Expression, and Purification of rLigA7’-13’ and rLigB1’-7’

**Figure 1:**
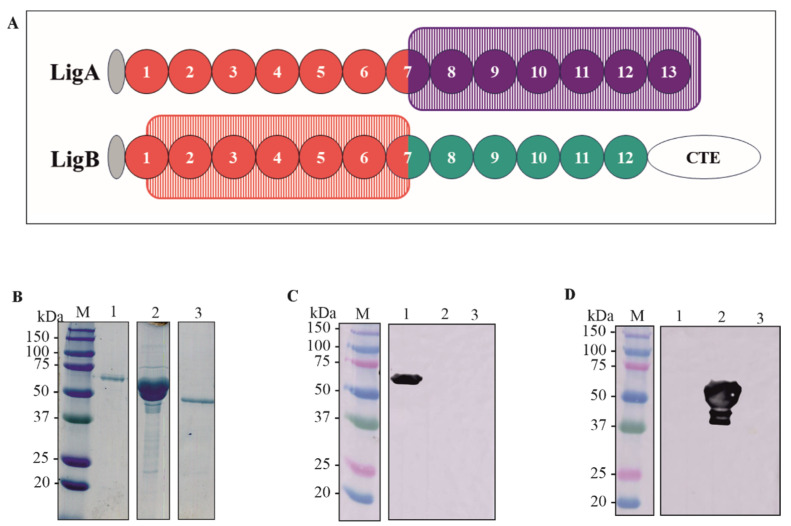
Schematic representation of the domains rLigA7’-13’ and rLigB1’-7’, protein purification and detection by polyclonal antibodies (**A**) A drawing showing the domains cloned of LigA7’-13’ (625-1224) in purple rectangle and LigB1’-7’ (131-645) in orange rectangle. (**B**) SDS-PAGE of purified rLigA7’-13’ and rLigB1’-7’ after dialysis against PBS. The lanes showed (M) molecular mass marker; (1) rLigA7’-13’ (2) rLigB1’-7’ (3) Lsa46 (control). (**C**,**D**) Western blotting of recombinant protein rLigA7’-13’, rLigB1’-7’, Lsa46 (control) revealed with polyclonal antibodies raised by immunization of BALB/c mice with each recombinant fragment. The lanes showed (M) molecular mass marker, (1) rLigA7’-13’, (2) rLigB1’-7’, and (3) Lsa46.

The sequences containing the domains of LigA7’-13’ (625–1224) and LigB1’-7’ (131–645) were amplified by PCR using genomic DNA from *L. interrogans* as a template. The drawing showing the selected domains is depicted in Figure 1A. The generated amplicons were cloned into the pET-28a vector at the NdeI-XhoI and SacI-XhoI restriction sites for rLigA7’-13’ and rLigB1’-7’, respectively. This vector adds a 6xHis tag to the N-terminal of the recombinant proteins. The corresponding DNA sequences were verified using an automated sequencer (3500 Genetic Analyzer, Applied Biosystems—Auburn, AL, USA).). The constructs pET-28a-LigA7’-13’ and pET-28a-LigB1’-7’ were used to transform *E. coli* C43 (DE3) and *E. coli* BL21 DE3, respectively, as these strains showed higher expression yields for each fragment. The recombinant proteins were expressed in their soluble form and purified using metal-chelating chromatography with an AKTA-prime system. The rLigA7’-13’ fraction was dialyzed against PBS supplemented with 2% glycine to prevent precipitation, while the rLigB1’-7’ fraction was dialyzed in PBS only. The proteins LigA7’-13’, rLigB1’-7’, and the control Lsa46 [34] were analyzed and quantified using SDS-PAGE. The expected molecular weights of 63 kDa (lane 1), 54 kDa (lane 2), and 46 kDa (lane 3) are shown in Figure 1B.

### 3.2. Detection of Recombinant Fragments by Western Blotting with the Respective Antiserum

Purified rLigA7’-13’ and rLigB1’-7 were administered to BALB/c mice in three doses, with a 14-day interval. Serum titration revealed that both fragments were immunogenic, with a titer of 4.0 × 10^5^ and 8.2 × 10^5^ for rLigA7’-13’ and rLigB1’-7, respectively. The recombinant proteins rLigA7’-13’, rLigB1’-7, and Lsa46, used as controls to verify cross-reactivity of polyclonal antibodies, were blotted onto nitrocellulose membrane and detected with anti-rLigA7’-13’ (1:5000) (Figure 1C) and anti-rLigB1’-7 (1:10,000) (Figure 1D) and HRP-conjugated anti-mouse IgG (1:1000). The bands showed expected molecular mass of 63 kDa to rLigA7’-13’ (lane 1) and 54 kDa to rLigB1’-7’ (lane 2) and no cross-reactivity with Lsa46 (control).

### 3.3. Binding of Recombinant Fragments to Human Integrins and to GAGs

**Figure 2:**
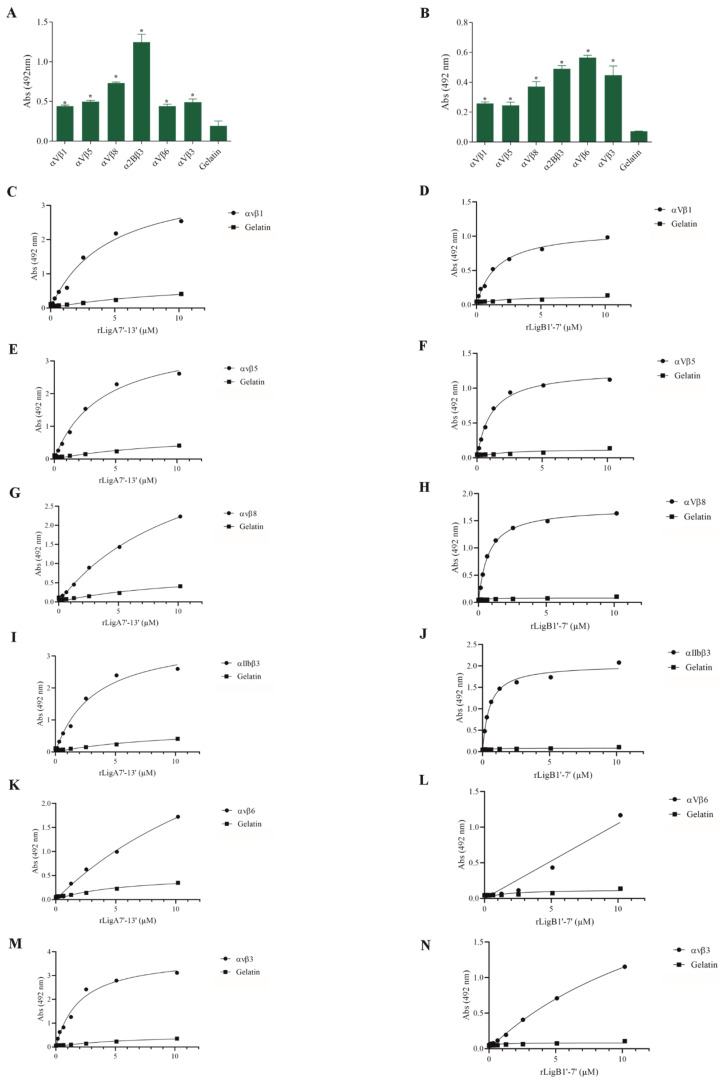
Recombinant proteins rLigA7’-13’ and rLigB1’-7’ interactions with RGD-binding integrins. (**A**) for rLigA7’-13’and (**B**) for rLigB1’-7’. ELISA plates were coated with RGD-binding integrins (100 nanograms) or gelatin (negative control). (**C**,**D**) αVβ1, (**E**,**F**) αVβ5 (**G**,**H**) αVβ8 (**I**,**J**) αIIBβ3 (**K**,**L**) αVβ6 and (**M**,**N**) αVβ3 were coated onto ELISA plates with different concentrations of rLigA7’-13’ or rLigB1’-7’ (0–10 µM) and detected with anti-rLigA7’-13’ (1:5000) or rLigB1’-7’ antiserum (1:10,000) and secondary HRP-conjugated anti-mouse IgG (1:5000). * Indicates significant difference calculated by two-tailed *t*-test with *p*-values < 0.05 (gelatin).

**Figure 3:**
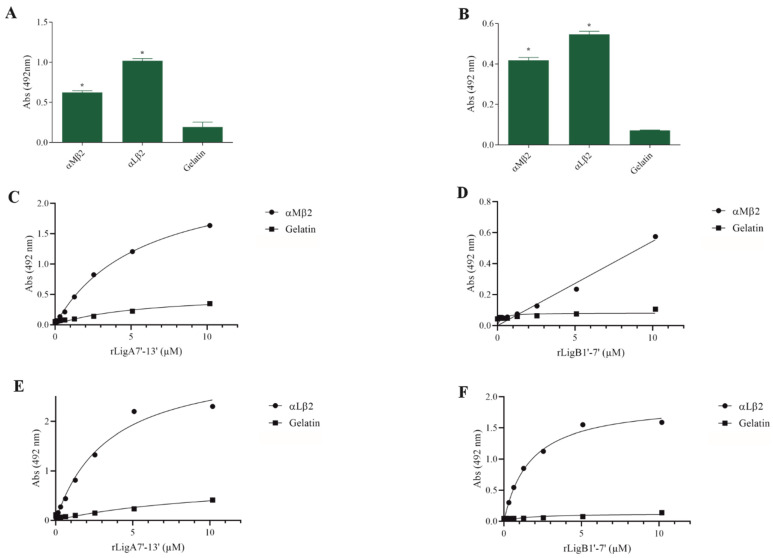
Binding of the recombinant proteins rLigA7’-13’ and rLigB1’-7’ to the leukocyte integrins. (**A**) for rLigA7’-13’and (**B**) for rLigB1’-7’. ELISA plates were coated with leukocyte integrins (100 nanograms) or gelatin (negative control). (**C**,**D**) αMβ1 and (**E**,**F**) αLβ2 were coated onto ELISA plates with different concentrations of rLigA7’-13’ or rLigB1’-7’ (0–10 µM) and detected with anti-rLigA7’-13’ (1:5000) or rLigB1’-7’ antiserum (1:10,000) and secondary HRP-conjugated anti-mouse IgG (1:5000). * Indicates significant difference calculated by two-tailed *t*-test with *p*-values < 0.05 (gelatin).

**Figure 4:**
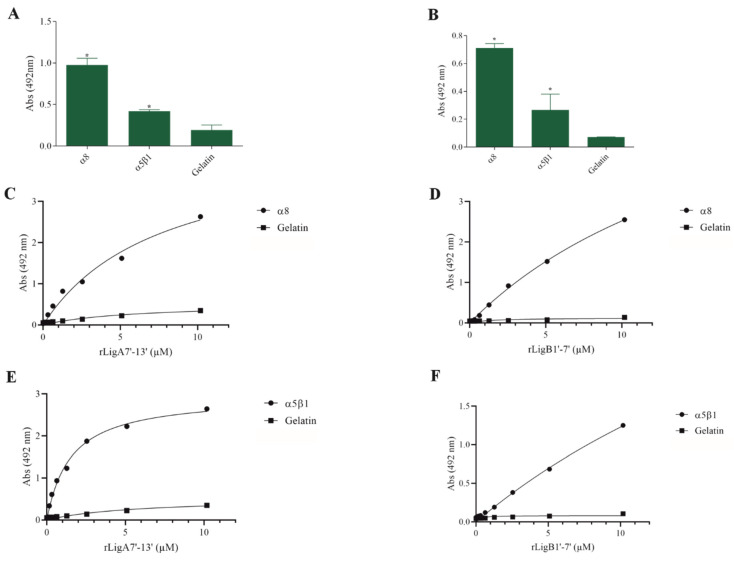
Recombinant proteins rLigA7’-13’ and rLigB1’-7’ bind to collagen and laminin integrins. (**A**) for rLigA7’-13’and (**B**) for rLigB1’-7’. ELISA plates were coated with collagen and laminin-binding integrins (100 nanograms) or gelatin (negative control). (**C**,**D**) α8 and (**E**,**F**) α5β18 were coated onto ELISA plates with different concentrations of rLigA7’-13’ or rLigB1’-7’ (0–10 µM) and detected with anti-rLigA7’-13’ (1:5000) or rLigB1’-7’ antiserum (1:10,000) and secondary HRP-conjugated anti-mouse IgG (1:5000). * Indicates significant difference calculated by two-tailed *t*-test with *p*-values < 0.05 (gelatin).

The interaction of pathogenic bacteria, including *Leptospira,* with integrins is crucial for adhesion to host cells [35,36,37]. We demonstrate that rLigA7’-13’ and rLigB1’-7’ significantly bind to RGD-binding integrins (αVβ1, αVβ5, αVβ8, αIIbβ3, αVβ6, and αVβ1) (Figure 2), leukocyte integrins (αMβ2 and αLβ2) (Figure 3), and collagen and laminin-binding integrins (α8 and α5β1) (Figure 4). All interactions were dose-dependent; however, for rLigB1’-7’ binding to αVβ6 (Figure 2L), αMβ2 (Figure 3D), α8, and α5β1 (Figure 4D,F), saturation was not reached. While both recombinant proteins exhibited a broad binding spectrum towards integrins, rLigA7’-13’ achieved a higher number of components with lower dissociation constants (K*_D_*), totaling 8, compared to 5 for rLigB1’-7’. Table 1 summarizes the K*_D_* of the binding reactions of recombinant proteins rLigA7’-13’ and rLigB1’-7’ to integrins.

**Figure 5:**
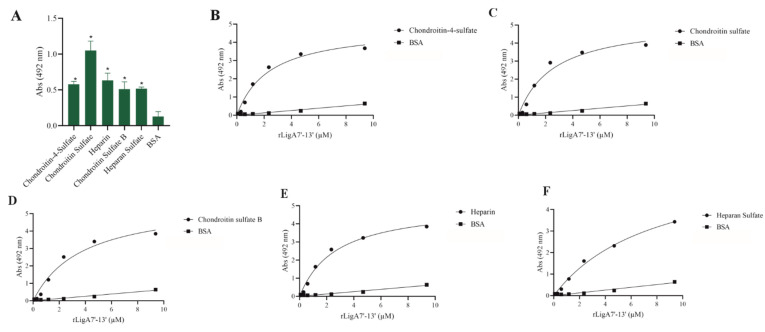
Evaluation of the recombinant protein rLigA7’-13’ binding to glycosaminoglycans. (**A**) Glycosaminoglycans were coated onto ELISA plates and incubated with rLigA7’-13’ (1 ug) for 2 h at 37 °C. Detection was performed with HRP-conjugated anti-HisTag mAbs (1:10, 000). (**B**) Coated chondroitin-4-sulfate, (**C**) chondroitin sulfate, (**D**) chondroitin sulfate B, (**E**) heparin, and (**F**) heparan sulfate were incubated with different concentrations of rLigA7’-13’ (0–10 mM) for 2 h at 37 °C and detected with HRP-conjugated anti-HisTag mAbs (1:10,000). BSA was used as negative control. * Indicates significant difference calculated by two-tailed *t*-test with *p*-values < 0.05 (BSA).

GAGs, the carbohydrate chains of proteoglycans, function as adhesion receptors for bacteria, viruses, and parasites [25]. Thus, we examined whether rLigA7’-13’ and rLigB1’-7’ could interact with these molecules. LigA7’-13’ showed affinity for chondroitin-4-sulfate, chondroitin sulfate, heparin, chondroitin sulfate B, and heparan sulfate, while LigB1’-7’ was unable to bind to any of the tested GAGs. The interaction of rLigA7’-13’ was dose-dependent with all these macromolecules (Figure 5), and the K_D_ values are shown in Table 1. These data suggest a higher plasticity of rLigA7’-13’ compared to LigB1’-7’ in relation to GAG interactions.

### 3.4. Interaction of rLigA7’-13’ and rLigB1’-7’ Proteins with the Terminal Complement System Molecules

**Figure 6:**
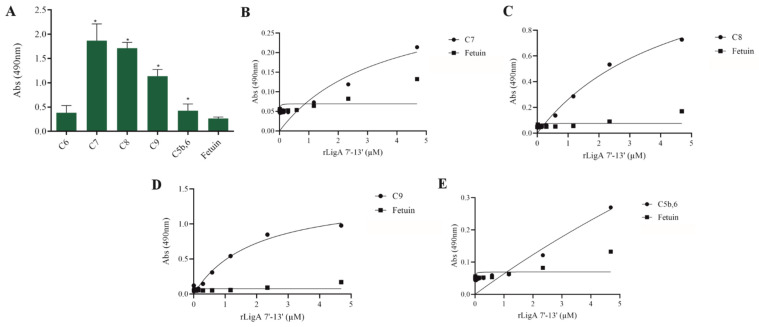
Evaluation of the recombinant protein rLigA7’-13’ binding with components of complement system. (**A**) Components were coated onto ELISA plates and incubated with rLigA7’-13’ (1 µg) for 2 h at 37 °C. Detection was performed with HRP-conjugated anti-HisTag mAbs (1:10,000). (**B**) Coated C7, (**C**) C8, (**D**) C9, and (**E**) C5b,6 were individually incubated with different concentrations of rLigA7’-13’ (0–5 µM) for 2 h at 37 °C and detected with HRP-conjugated anti-HisTag mAbs (1:10,000). Fetuin was used as negative control. * Indicates significant difference calculated by Student *t*-test with *p*-values < 0.05 (fetuin).

**Figure 7:**
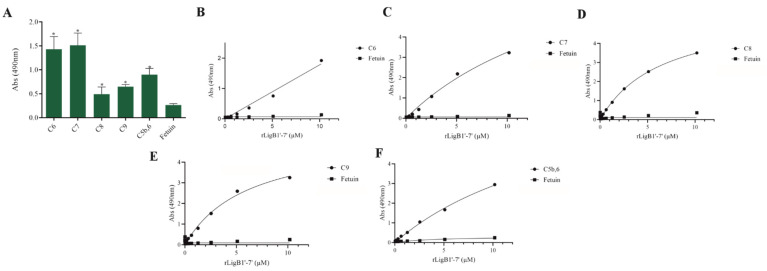
Evaluation of the recombinant protein rLigB1’-7’ binding with complement system components. (**A**) Components were coated onto ELISA plates and incubated with rLigB1’-7’ (1 µg) for 2 h at 37 °C. Detection was performed with HRP-conjugated anti-HisTag mAbs (1:10,000). (**B**) Coated C6, (**C**) C7, (**D**) C8, (**E**) C9 (**F**) C5b,6 were individually incubated with different concentrations of rLigA7’-13’ (0–5 µM) for 2 h at 37 °C and detected with HRP-conjugated anti-HisTag mAbs (1:10,000). Fetuin was used as negative control. * Indicates significant difference calculated by two-tailed *t*-test with *p*-values < 0.05 (Fetuin).

**Figure 8:**
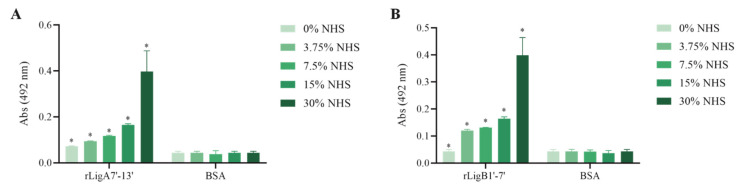
C9 uptake from NHS by the recombinant proteins. (**A**) rLigA7’-13’ and (**B**) rLigB1’-7’ were immobilized and incubated with increasing concentrations of NHS. The interaction was assessed by the addition of each goat anti-human complement component (1:10,000), followed by HRP-conjugated anti-goat IgG (1:50,000). BSA was used as negative control. * Indicates significant difference calculated by two-tailed *t*-test with *p*-values < 0.05 (BSA).

The interaction, capture, and degradation of complement system proteins by pathogenic *Leptospira* can help the bacteria escape the host’s innate immune system and prolong infection by inhibiting the formation of the membrane attack complex (MAC) [38]. rLigA7’-13’ exhibited a higher affinity for C7, C8, and C9, while lower binding affinity was observed with C6 and C5b,6 (Figure 6). The protein rLigB1’-7’ demonstrated significant affinity for all tested components (Figure 7). The dose–response of both recombinant proteins was concentration-dependent and saturable, except for the interaction of rLigA7’-13’ with C5b,6 (Figure 6E) and the interactions of rLigB1’-7’ with C6 and C5b,6 (Figure 7B,F). To investigate whether the recombinant proteins could acquire C9 from NHS, immobilized proteins were reacted with increasing percentages of NHS (3.75% to 30%), and the reaction was detected with anti-C9. The data show that the binding of both proteins to C9 intensified with increasing serum concentration (Figure 8A,B).

**Figure 9:**
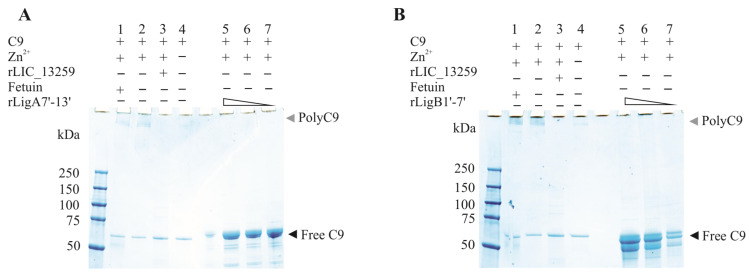
Zinc-induced C9 polymerization inhibition by rLigA7’-13’ (**A**) and rLigB1’-7 (**B**)’. C9 polymerization was assessed by incubating 3 µg of C9 with fetuin (negative inhibition control, lane 1), C9 plus zinc (lane 2-positive control), 5 µg of either rLIC_13259 (positive inhibition control, lane 3), C9 only (control—lane 4), or decreasing amounts of rLigA7’-13’ (panel A, lanes 5–7) and rLigB1’-7’ (panel B, lanes 5–7). The mixtures were incubated at 37 °C for 30 min, followed by the addition of ZnCl₂ to a final concentration of 50 µM and further incubation for 2 h at 37 °C. Polymerization controls included C9 alone with (lane 2) or without (lane 4) ZnCl_2_. The reactions were analyzed using 4–20% gradient SDS-PAGE and visualized with Coomassie blue staining.

To evaluate whether the interaction of rLigA7’-13’ and rLigB1’-7 with C9 inhibits poly-C9 formation, C9 was incubated with increasing amounts of rLigA7’-13’ and rLigB1’-7’, with polymerization induced by the addition of Zn^2+^ to the reaction mixture. As controls, one or more components were omitted, and fetuin and rLIC_13259 were used as negative and positive controls, respectively. The reaction mixtures were subjected to gradient SDS-PAGE, and protein bands were revealed by Coomassie blue staining. Increasing the protein concentration from 1.25 to 5 µg of rLigA7’-13’ or rLigB1’-7’ decreased poly-C9 formation (Figure 9A,B). Thus, it is possible that leptospiral immune evasion promoted by rLigA7’-13’ and rLigB1’-7’ proteins occurs through two different mechanisms: either by binding to complement regulators [24] or by interacting with the terminal components of the complement system.

## 4. Discussion

The successful promotion of an infectious disease involves the processes of adhesion to the host, tissue invasion, and evasion or overcoming of the host immune defenses [39]. Thus, the ability to adhere to host cells and the ECM is a crucial aspect of the infectious process for many pathogens. Adhesion is likely vital for *Leptospira,* but the mechanisms they use for adhesion during infection have not been studied as extensively as those of other bacterial pathogens.

LigA and LigB are unique to pathogenic *Leptospira* and are among the most researched proteins [13,20,40,41,42,43,44]. They mediate interactions with the ECM, plasma proteins, and components of the complement system [19,20,22,45]. In this study, we report the binding of rLigA7’-13’ and rLigB1’-7’ domains to integrins, GAGs, and components of the terminal pathway of the complement system—interactions that have not been investigated before. The LigA7’-13’ and LigB1’-7’ domains were expressed in *E. coli* as soluble proteins and efficiently purified. Both proteins proved to be immunogenic, resulting in high antibody titers following immunization in mice.

GAGs, the carbohydrate chains found in proteoglycans, are prevalent in animal cells and, as discussed below, are common targets for bacterial binding [25,26]. Numerous pathogens express surface proteins that recognize GAGs, including *Neisseria gonorrhoeae* [46], *Bordetella pertussis*, and *Mycobacteria* spp. [47], *Listeria monocytogenes* [48], and enterotoxigenic *E. coli* [49]. The spirochete *Borrelia burgdorferi* expresses GAG-binding adhesins such as DbpA, DbpB, and BBK32, all of which facilitate colonization of mammalian hosts [50,51,52,53]. Additionally, OspF-related proteins have been identified, at least one of which promotes bacterial attachment to glial cells [54]. The interaction between pathogenic leptospires and GAGs has been documented, with leptospiral proteins identified as GAG-binding proteins [28,29,32,55]. The LigA7’-13’ domain exhibited a broad binding spectrum to GAGs, interacting with chondroitin-4-sulfate, chondroitin sulfate, heparin, chondroitin sulfate B, and heparan sulfate. In contrast, no binding was detected for LigB1’-7’. These findings suggest that the LigA7’-13’ domain has greater interaction plasticity compared to LigB1’-7’.

Integrins are crucial cell adhesion receptors involved in various biological processes through their binding to different ligands [24]. Many of these interactions occur via the RGD motif found in ECM components. Leptospiral proteins Mce and the LRR protein LIC_11505 have been reported to interact with several integrins [32,56]. The *mce^-^* mutant demonstrated significantly reduced efficiency in infecting hamsters compared to the wild-type *L. interrogans* strain [56]. Both LigA7’-13’ and LigB1’-7’ show substantial interactions with RGD-binding integrins, leukocyte integrins, and collagen- and laminin-binding integrins. However, integrins are complex components that have specialized functions. It is speculated that the binding of cell adhesion molecules may initiate bacterial incorporation, leading to cytoskeletal rearrangements triggered by receptor assembly [57]. Therefore, the interactions of recombinant LigA7’-13’ and LigB1’-7’ with various integrins require further investigation.

Pathogens evolved a range of strategies to bypass the innate immune response and successfully establish infections in human hosts. Notably, research from years ago highlighted that only pathogenic strains of *Leptospira* could withstand the bactericidal effects of non-immune human serum [58]. Further investigations revealed that both fully and partially resistant strains were isolated from human patients, while the saprophyte *L. biflexa* was identified as the most susceptible. The mechanism behind this killing was linked to the complement system, with resistant strains showing an enhanced ability to bind FH compared to their more vulnerable counterparts [59].

In subsequent studies, it became clear that pathogenic *Leptospira* can acquire soluble human complement regulators, including FH, FH-related protein 1, and C4BP [60,61]. Many pathogenic microorganisms have evolved sophisticated mechanisms to bind FH, leveraging its properties to evade lysis mediated by complement. Proteins interacting with complement regulators have been discovered in spirochetes such as *Borrelia* and *Treponema*, including the FhbB protein from *Treponema denticola* and OspE and CRASPs from *B. burgdorferi* [62,63]. Pathogenic *Leptospira* have been found to express protein receptors for FH and C4BP, such as LfhA/Lsa24/LenA [61] and the LigA7’-13’, LigB1’-7’, and LigB7’-13’ proteins [22]. Recent findings indicate that pathogenic *Leptospira* are capable of binding various complement-regulatory proteins, including FH, FHL-1, FHR-1, C4BP, vitronectin, and several terminal complement proteins [38]. Notably, LigA7’-13’ and LigB1’-7’ interact with terminal complement proteins and have been shown to recruit C9 from NHS. This interaction may hinder the formation of polyC9, thereby increasing the release of free C9 and ultimately preventing the assembly of the MAC. Thus, it appears that LigA7’-13’ and LigB1’-7’ may play a significant role in inhibiting MAC formation in two possible ways: by interacting with complement regulators of classical and alternative pathways [22] and/or by interfering directly with terminal complement components.

In conclusion, this study demonstrates that the LigA7’-13’ and LigB1’-7’ domains of the respective proteins not only interact with various extracellular matrix (ECM) components and host cell receptors, as previously reported, but also exhibit binding affinity for GAGs, integrins, and components of the terminal complement system. These findings expand the understanding of their significant roles in leptospiral virulence.

## Figures and Tables

**Table 1:** Dissociation constants (K*_D_*) of the recombinant proteins binding to integrins, GAGs, and complement system components (μM).

Host	Components	rLigA7’-13’ (μM)	rLigB1’-7’ (μM)
Integrins	αVβ1	4.45 ± 2.82	1.57 ± 0.12
	αVβ5	3.84 ± 1.91	1.09 ± 0.31
	αVβ8	11.36 ± 1.33	0.72 ± 0.02
	αIIbβ3	3.25 ± 0.24	0.50 ± 0.005
	αVβ6	20.39 ± 8.31	31.00 ± 18.69
	αVβ3	1.98 ± 2.12	16.88 ± 0.77
	αMβ2	5.69 ± 1.18	72.00 ± 23.93
	αLβ2	3.44 ± 1.14	1.68 ± 0.66
	α8	7.66 ± 4.58	19.45 ± 8.22
	α5β1	1.51 ± 2.75	34.22 ± 12.95
GAGs	Chondroitin-4-sulfate	2.38 ± 0.47	-
	Chondroitin sulfate	2.62 ± 0.77	-
	Chondroitin sulfate B	3.78 ± 0.93	-
	Heparin	2.70 ± 0.52	-
	Heparan Sulfate	7.98 ± 4.87	-
Complement system components	C5b,6	ND	ND
	C6	-	ND
	C7	3.59 ± 2.03	18.78 ± 4.92
	C8	4.62 ± 4.12	6.50 ± 6.12
	C9	1.95 ± 3.88	6.12 ± 5.72

ND: Not Determined.

## Data Availability

The original contributions presented in this study are included in the article. Further inquiries can be directed to the corresponding author.
